# Structural characterization of coatomer in its cytosolic state

**DOI:** 10.1007/s13238-016-0296-z

**Published:** 2016-07-29

**Authors:** Shengliu Wang, Yujia Zhai, Xiaoyun Pang, Tongxin Niu, Yue-He Ding, Meng-Qiu Dong, Victor W. Hsu, Zhe Sun, Fei Sun

**Affiliations:** 1 National Key Laboratory of Biomacromolecules, Institute of Biophysics, Chinese Academy of Sciences, Beijing, 100101 China; 2 National Institute of Biological Sciences, Beijing, Beijing, 102206 China; 3 Center for Biological Imaging, Institute of Biophysics, Chinese Academy of Sciences, Beijing, 100101 China; 4 University of Chinese Academy of Sciences, Beijing, 100049 China; 5 Department of Medicine, Harvard Medical School, Brigham and Women’s Hospital, Boston, MA 02115 USA

**Keywords:** coatomer, COPI, human, single-particle electron microscopy, membrane trafficking

## Abstract

Studies on coat protein I (COPI) have contributed to a basic understanding of how coat proteins generate vesicles to initiate intracellular transport. The core component of the COPI complex is coatomer, which is a multimeric complex that needs to be recruited from the cytosol to membrane in order to function in membrane bending and cargo sorting. Previous structural studies on the clathrin adaptors have found that membrane recruitment induces a large conformational change in promoting their role in cargo sorting. Here, pursuing negative-stain electron microscopy coupled with single-particle analyses, and also performing CXMS (chemical cross-linking coupled with mass spectrometry) for validation, we have reconstructed the structure of coatomer in its soluble form. When compared to the previously elucidated structure of coatomer in its membrane-bound form we do not observe a large conformational change. Thus, the result uncovers a key difference between how COPI versus clathrin coats are regulated by membrane recruitment.

**Electronic supplementary material:**

The online version of this article (doi:10.1007/s13238-016-0296-z) contains supplementary material, which is available to authorized users.

## Introduction

Coat proteins play a key role in intracellular transport by initiating the formation of transport vesicles. They achieve this role by coupling two major functions, bending membrane that generates transport carriers from organellar membrane, and binding to cargoes for the proper packaging of newly formed vesicles. The ADP-ribosylation factor (ARF) family of small GTPases has been shown to regulate these function of coat proteins by dictating their recruitment from the cytosol to membrane (Bonifacino and Glick, 2004).

Studies on coat protein I (COPI) have contributed to a basic understanding of how coat proteins achieve its roles in vesicle formation and cargo sorting (Faini et al., 2013; Hsu et al., 2009; Jackson, 2014; Pucadyil and Schmid, 2009). The core component of the COPI complex is coatomer, which exists as a multimeric complex containing seven subunits: α-, β-, β′-, γ-, δ-, ε-, and ζ-COP (Hara-Kuge et al., 1994; Malhotra et al., 1989; Waters et al., 1991). Large efforts have been made to elucidate the structure of coatomer over the years, including several crystal structures of fragments of the seven subunits (Jackson et al., 2012; Lee and Goldberg, 2010; Ma and Goldberg, 2013; Suckling et al., 2015; Yu et al., 2012), and electron microscopy (EM)-based reconstruction of the entire structure of coatomer (Dodonova et al., 2015; Yip and Walz, 2011).

Coatomer can be further divided into two subcomplexes, with the B-subcomplex containing α, β′, and ε subunits and the F-subcomplex containing β, δ, γ, and ζ subunits. The F-subcomplex shows significant structural similarity to the clathrin adaptors and interacts with two molecules of activated (GTP-bound) form of ARF1 (ADP-ribosylation factor 1) via the N-termini of β- and γ-COP (Faini et al., 2013; Serafini et al., 1991b; Yu et al., 2012). In the B-subcomplex, α- and β′-COP are considered structural homologs. Both adopt the domain structure of β-propeller-β-propeller-α-solenoid, and they form a heterodimer via the α-solenoid region (Lee and Goldberg, 2010).

In recent years, structural studies on the clathrin adaptors have shed important additional insights into how membrane recruitment regulates the function of coat proteins. Clathrin AP2 has been found to undergo a major conformational change upon its recruitment from the cytosol to membrane (Jackson et al., 2010). This change is instigated by AP2 binding to a key lipid, phosphatidylinositol (4,5) bis-phosphate (PIP2), at the plasma membrane. The soluble form of AP2 exists in a “closed” conformation, which prevents cargo binding by AP2 (Collins et al., 2002). Binding to PIP2 on membrane induces an “open” conformation of AP2 to allow cargo binding (Jackson et al., 2010). Structural study on clathrin AP1 also suggests that it undergoes a major conformational change upon membrane recruitment (Ren et al., 2013). In the soluble state, AP1 adopts a similarly “closed” conformation to prevent cargo binding (Heldwein et al., 2004). Upon binding to ARF1 on membrane, AP1 shifts into an “open” conformation to allow cargo binding (Ren et al., 2013).

As basic mechanisms of intracellular transport are generally conserved (Bonifacino and Glick, 2004), coatomer is predicted to undergo a similar large conformational change during membrane recruitment. However, recent structural reconstructions of membrane-bound coatomer have begun to identify key differences between how COPI and the clathrin complexes are organized on membrane for vesicle formation (Dodonova et al., 2015; Faini et al., 2012). As such, whether the large conformational changes that have been observed for the membrane recruitment of clathrin adaptors would also apply to the COPI complex becomes an open question. In this study, we address this key mechanistic issue by elucidating the structure of coatomer in its soluble form. By comparing this structure with the previously solved structure of coatomer in its membrane-bound form (Dodonova et al., 2015), we have uncovered yet another key difference between coatomer and the clathrin adaptors.

## Results

### Expression and purification of recombinant human coatomer

**Figure 1:**
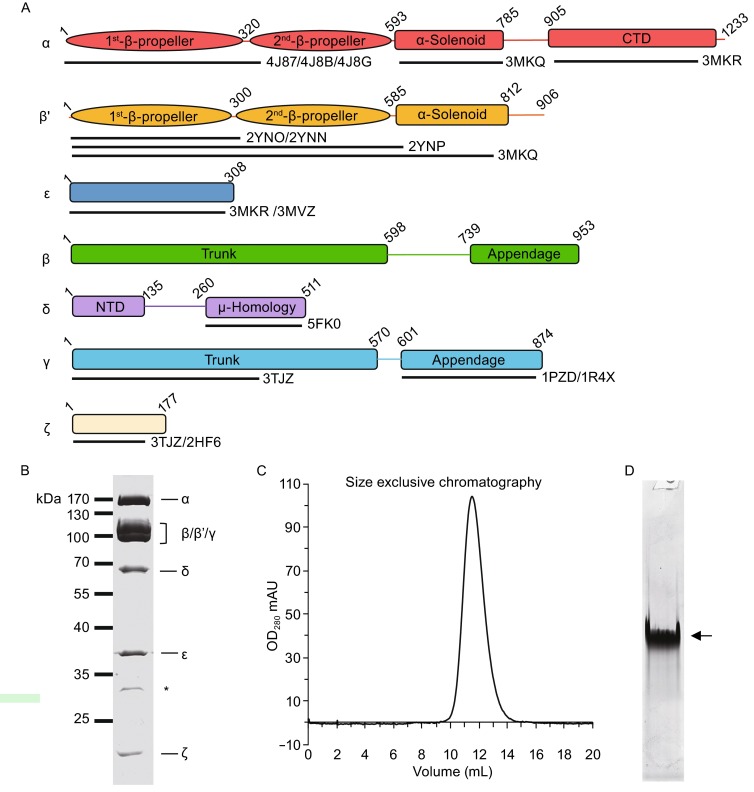
**Preparation of purified recombinant human coatomer**. (A) Schematic diagram showing the domain organization of each subunits of human coatomer. The available atomic structures with their PDB IDs and sequence coverage are also labeled. (B) Coomassie-blue stained SDS-PAGE of recombinant coatomer with all the subunits labeled. Asterisk indicates the N-terminal fragment of δ-COP, which is confirmed by mass spectrometry and Western blot. (C) Gel filtration of purified coatomer. The retention volume of the single peak on the column of Superose 6 (GE healthcare) is 11.4 mL. (D) Native PAGE of the purified coatomer. Arrow indicates the intact coatomer

Coatomer exists as a single complex of approximately 560 kDa (Waters et al., 1991). The subunits of coatomer and their major domains are shown in Fig. 1A. To facilitate the isolation of highly purified coatomer for structural analysis, recent studies have generated recombinant mouse coatomer using a MultiBac expression system (Dodonova et al., 2015; Faini et al., 2012; Sahlmuller et al., 2011). In the present study, we developed a novel protein complex expression system, ViperTEC (to be published elsewhere), to express recombinant human coatomer in insect cells. Specifically, cDNAs of α-, β′-, and ε-COP were inserted into one expression cassette containing a p6.9 promoter and a SV40 terminator, while cDNAs for the other four subunits (β-, γ-, δ-, and ζ-COP) were inserted into another expression cassette containing a p10 promoter and a SV40 terminator. Based on previous structural studies (Jackson et al., 2012; Lee and Goldberg, 2010; Ma and Goldberg, 2013; Suckling et al., 2015; Yu et al., 2012), tags were introduced at the 5′ end of each subunit to minimize potential effects on the subsequent structure and function of coatomer. As will be seen below, this has facilitated our ability to track the expression of individual subunits and their assembly state during purification. Moreover, we did not detect significant effects on coatomer function.

To purify the recombinant human coatomer expressed in insect cells, we performed Strep tag II affinity chromatography followed by Mono Q anion exchange chromatography. Multiple observations suggested that this approach resulted in intact coatomer being purified. First, Coomassie-stained SDS-PAGE gel showed the presence of all seven subunits of coatomer (Fig. 1B), which was further verified by Western blotting (Fig. S1). Second, gel filtration showed a single peak during elution (Fig. 1C). Third, native PAGE reveals a mono-disperse band, indicating that the recombinant coatomer was isolated with high purity and homogeneity (Fig. 1D). Fourth, analysis using liquid chromatography mass spectrometry (LC-MS) of the single band observed in native page resulted in all seven subunits of coatomer being identified (Table 1). Our yield of purified coatomer was approximately 1.5 mg per liter of cell culture.

**Table 1:** Mass spectrometry analysis of purified coatomer extracted from the band of native PAGE

Subunit	Molecular mass (kDa)	Unique peptide (n)	Coverage (%)
α-COP	139	108	77.5
β-COP	107	84	68.5
β′-COP	102	64	74.2
γ-COP	98	69	74.6
δ-COP	57	33	54.2
ε-COP	34	22	83.4
ζ-COP	20	6	37.9

Our previous experiences in purifying native coatomer from tissue suggested that the linkage between the μ-homology domain and the N-terminal domain (NTD) of δ-COP is flexible, as the μ-homology domain was frequently degraded (unpublished data). In the purification of recombinant coatomer, we also observed the degradation of δ-COP, which appeared as an additional band in Coomassie-stained gel (Fig. 1B). Western blotting (Fig. S1) and mass spectrometry analysis (Table S1) identified this additional band as the N-terminal portion of δ-COP. Collectively, these observations suggested that a portion of δ-COP is likely to be highly exposed on the surface of coatomer, as this portion shows consistent partial degradation during the purification of coatomer.

### *In vitro* functional assays of the recombinant human coatomer

**Figure 2:**
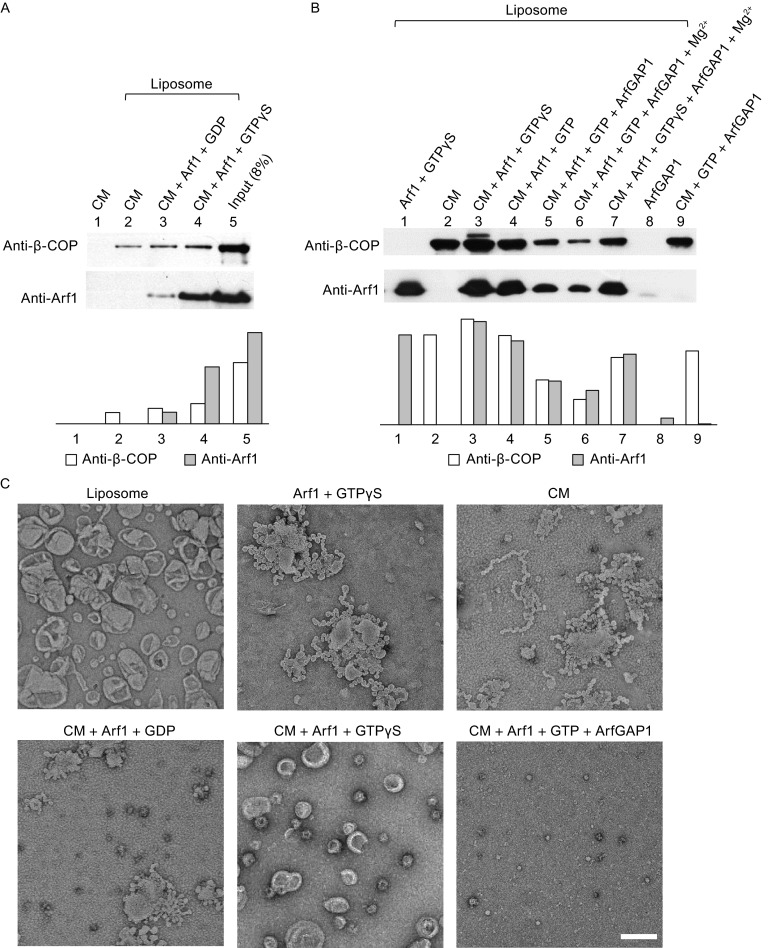
**Membrane binding and vesiculation assays**. (A and B) Membrane binding co-sedimentation assays (See Materials and methods). The amount of co-sedimented coatomer (β-COP) and ARF1 is also quantified in lower panels. (C) Negative-stain electron microscopy of liposomes incubated with coatomer and other factors. Scale bar, 200 nm. CM, human coatomer; ARF1, N-myristoylated human ARF1; ARFGAP1, rat ARFGAP1

We next assessed whether the purified recombinant human coatomer is functional. For this goal, we generated recombinant human N-myristoylated ARF1 (Fig. S2A). Moreover, we used liposomal membrane rather than Golgi membrane, as native membrane contains a myriad of other proteins that can confound the ability to assess directly the roles of ARF1 and coatomer in COPI vesicle formation. When recombinant forms of ARF1 and coatomer were incubated with liposomes that contain a Golgi-like composition of pure lipids, we confirmed that ARF1 enhanced the recruitment of coatomer onto membrane (Fig. 2A). We also confirmed that the GTP-bound form of ARF1 was more efficient in binding to liposomes and in recruiting coatomer to this membrane (Fig. 2A). Thus, the recombinant forms of ARF1 and coatomer that we had generated are functional with respect to membrane recruitment.

We next considered that the GTPase-activating protein (GAP) responsible for catalyzing the deactivation of ARF1 during COPI vesicle formation is ARFGAP1 (Cukierman et al., 1995; Yang et al., 2002). Thus, we also purified the recombinant form of ARFGAP1 (Fig. S2B). Consistent with previous studies that have found ARFGAP1 to induce the release ARF1 from Golgi membrane (Cukierman et al., 1995; Yang et al., 2002), we found that our recombinant ARFGAP1 also reduced the level of ARF1 on liposomal membrane (Fig. 2B). This effect of ARFGAP1 could be attributed to its catalytic activity, as the use of GTPγS (a non-hydrolyzable GTP analogue) prevented the ability of ARFGAP1 to reduce the level of ARF1 on liposomal membrane (Fig. 2B).

**Figure 3:**
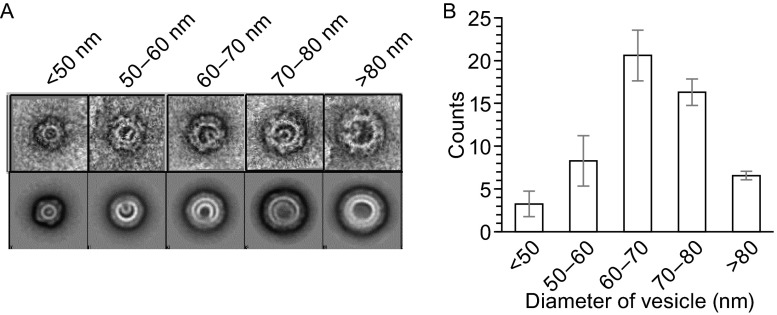
**Diameter distribution of reconstituted COPI-coated vesicles**. (A) The raw images of vesicles (first row) and the corresponding 2D class averages of vesicles with the same diameter (second row). The diameters are indicated on the top. (B) A statistical histogram of the vesicle number vs. different diameters. All error bars represent standard deviation (s.d.) from three independent vesicle reconstitution experiments. For each experiment, the number (N) of micrographs is 90

We next examined the effect of recruiting different combinations of coatomer, ARF1, and ARFGAP1 on membrane morphogenesis. ARF1 has been found previously to induce membrane curvature by inserting its N-terminal amphipathic helix into the membrane, which is reflected by the generation of tubules that sometimes also contain constrictions, resulting in a “beads-on-string” morphology (Beck et al., 2008; Krauss et al., 2008; Lundmark et al., 2008). We observed similar effects when recombinant ARF1 was incubated with Golgi-like liposomes (Fig. 2C). As the co-incubation of activated ARF1 and coatomer has been found previously to be sufficient to produce COPI vesicles from liposomal membrane (Bremser et al., 1999; Spang et al., 1998), we also confirmed this finding using the recombinant forms of ARF1 and coatomer that we had generated (Fig. 2C). Further scrutiny of these vesicles revealed that they are coated (Fig. S3A) and have diameter in the range of 50–80 nm (Fig. 3). Western blotting confirmed the presence of ARF1 and β-COP on these vesicles (Fig. S3B).

We also noted that COPI vesicles reconstituted from Golgi membrane have been found to be relatively depleted of cholesterol (Brugger et al., 2000). Consistent with this previous finding, we found that liposomal membrane was more readily vesiculated with lower concentrations of cholesterol (Fig. S4A, and also Supplemental text). The diameters of vesicles were also affected by the level of cholesterol (Fig. S4B, and also Supplemental text).

Besides ARF1 and coatomer, ARFGAP1 has been found previously to promote COPI vesicle formation (Yang et al., 2002). Consistent with this finding, we observed vesiculation when ARFGAP1 was added in conjunction with ARF1 and coatomer to liposomes (Fig. 2C). In this case, vesicles of smaller diameter (in the range of 30 nm) were observed (Fig. 2C). Similar to ARF1, ARFGAP1 is also known to possess a domain that can insert into the membrane (Bigay et al., 2003). Thus, a likely explanation is that the combination of ARF1, coatomer, and ARFGAP1 results in greater membrane curvature being generated, which is manifested by the formation of smaller vesicles.

We also found that coatomer alone could be recruited onto liposomal membrane (Fig. 2B), with EM examination revealing that this condition resulted in liposome tubulation (Fig. 2C). To our knowledge, this finding revealed a property of coatomer that had not been widely appreciated. Future studies will be needed to elucidate more precisely how coatomer exerts this effect. In any case, as the cumulative results above represent substantial support that the recombinant form of human coatomer that we have generated is functional, we next pursued structural analysis of this coatomer in its soluble form.

### Electron microscopy of coatomer in its soluble form

We initially pursued cryo-electron microscopy but failed to obtain high-quality micrographs, which could be attributed to coatomer being prone to collapse after cryo-vitrification, as the air-water interface that occurs in this procedure could produce damaging effects. Thus, we next pursued negative-stain electron microscopy coupled with computational image-processing methods.

We detected soluble coatomer as mono-dispersed particles with diameter in the range of 13–20 nm (Fig. S5A). These were selected for further image processing. Using reference-free 2D classification, we found that class averaging of most particles revealed a central density that is relatively invariant (Fig. S5B), while more peripheral densities appear variable in both location and shape (Fig. S5B). These findings were consistent with previous observations on the structure of yeast coatomer (Yip and Walz, 2011). Further scrutiny of the central density revealed that it exists as two parts (Fig. S5B and S5C). We also deduced that the variable extra densities could be attributed to flexible domains known to exist in coatomer. Besides δ-COP, these would include portions of the α^CTD^/ε-COP heterodimer and the appendage domains of β/γ-COP.

**Figure 4:**
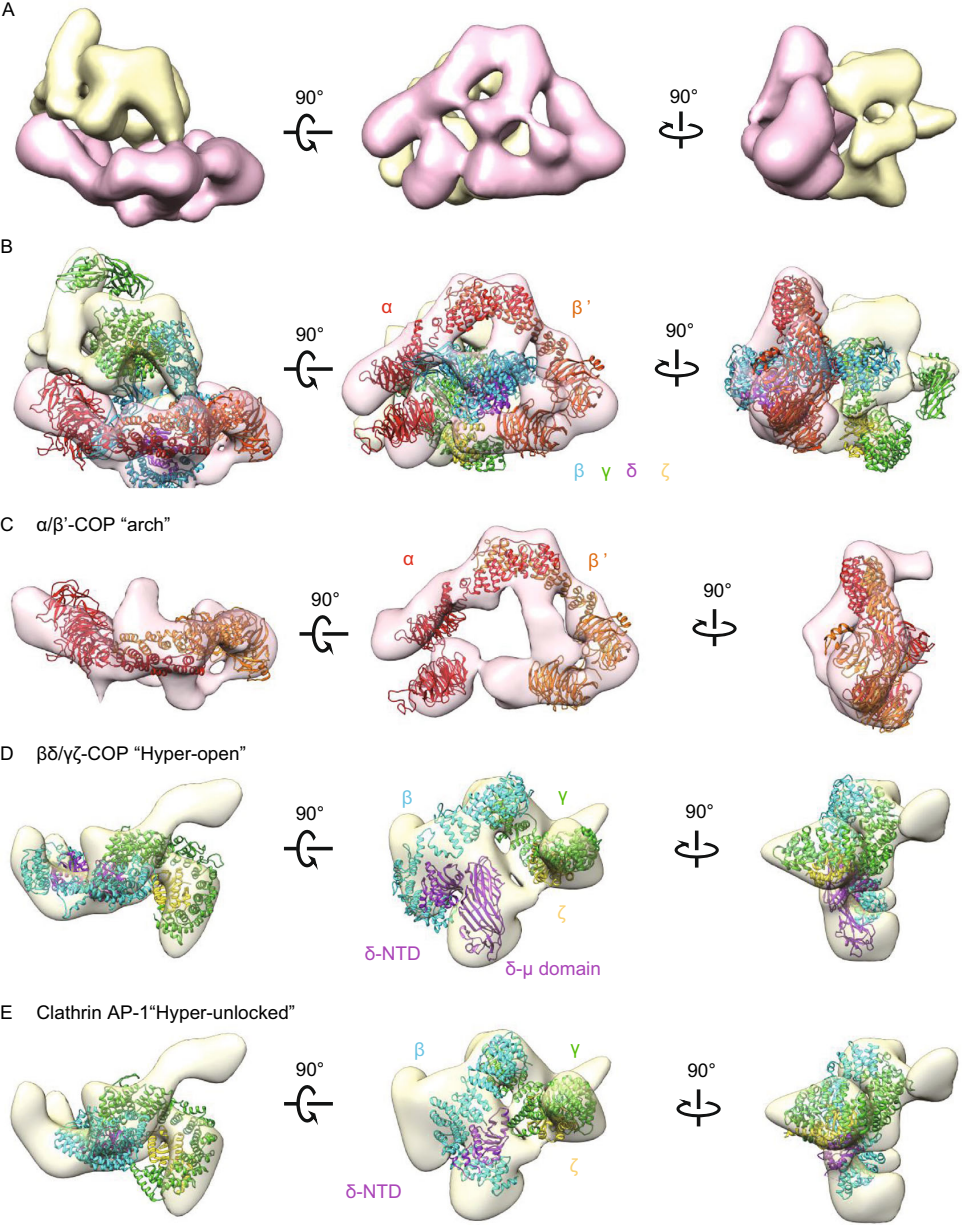
**3D reconstruction of the recombinant human coatomer in its soluble form**. (A) Three views of surface-rendered density map of coatomer obtained by single-particle reconstruction. The map is segmented using UCSF Chimera, grouped, and colored with pink for the arch-shape portion and yellow for the more globular portion. (B) Fitting the membrane-bound model of coatomer (PDB ID: 5A1U) into the EM map. (C) Fitting the B-subcomplex αβ′-COP heterodimer into the “focus” refined arch-shape density map. (D) Fitting the F-subcomplex βδ/γζ-COP into the “focus” refined globular density map. (E) Fitting the homologue model of F-subcomplex in “hyper-unlocked” form into the “focus” refined globular density map. α-, β′-COP are colored in red and orange. β-, δ-, γ-, and ζ-COP and their homologues are colored in cyan, purple, green, and yellow respectively

We then pursued single-particle analysis. To avoid potential modeling bias, we collected the tilt series of negatively stained coatomer and performed electron tomographic reconstruction followed by sub-tomogram averaging to generate a reliable model. We performed intensive iterative 2D and 3D classifications to sort the particle sets. We filtered out overlapping, imperfectly stained, or disrupted particles. The final reconstruction based on the filtered dataset yielded a 3D structure of coatomer with a resolution of 19 Å, as judged by Fourier shell correlation (FSC) criterion of 0.143 (Rosenthal and Henderson, 2003) (Figs. 4A and S5). Consistent with the results from the 2D class averages, the overall reconstructed EM map revealed soluble coatomer to possess two relatively independent parts. One part (part 1) adopts an arch shape (Fig. 4A, pink), while another part (part 2) sits adjacent to the arch-shaped structure and appears more globular (Fig. 4A, yellow). The arch-shaped density (part 1) was invariant during iterative 3D classifications, while the globular density (part 2) varied in different cycles of 3D classifications.

As the two parts of coatomer appeared to behave differently and independently, we next pursued local optimization (Shan et al., 2016) and performed “focus” refinement (Bai et al., 2015) to reconstruct the two parts separately (Fig. S6A; see also “MATERIALS AND METHODS”). The “focus” refinement resulted in a similar density map for the arch-shaped portion (part 1), and a different density map for the globular portion (part 2) (Fig. S6A). The final resolution was 18 Å and 20 Å, respectively, with FSC of 0.143 (Rosenthal and Henderson, 2003) (Fig. S6B). There was a flexible oscillation between the two parts, and the alignment procedure preferred the more stable part1 during the conventional refinement (Figs. 4C and S5A).

The recently solved structure of membrane-bound coatomer (Dodonova et al., 2015) provided an opportunity to analyze and interpret the EM maps of the soluble coatomer that we have generated. In order to detect potential structural difference between the two states, we initially fitted the complete structural model of the membrane-bound coatomer (PDB ID: 5A1U) as a rigid body onto the integrated density map before the “focus” refinement using UCSF *Chimera* (Pettersen et al., 2004). A good match was observed between the α/β′-COP heterodimer and the arch-shaped density. Thus, we assigned the arch-shaped density to correspond to the B-subcomplex of coatomer (Fig. 4B). In further support of this assignment, we noted that the α/β′-COP heterodimer has been suggested to be rigid, showing little difference when comparing the structure in solution (Lee and Goldberg, 2010) versus that on membrane (Dodonova et al., 2015). Thus, when taken collectively, we also assigned the other part of coatomer that appears as a globular density to correspond to the F-subcomplex. However, the fit in this case was relatively weak (Fig. 4B). This could be attributed to conformational changes of F-subcomplex between two states, or the potential flexible oscillation between the two subcomplexes of coatomer.

With the improved density map after “focus” classification and refinement, we also compared our reconstructed F-subcomplex with the three crystal structures of clathrin AP1 (the F-subcomplex homologue) in different conformations as well as the structure of membrane-bound F-subcomplex in the “hyper-open” state (Dodonova et al., 2015) to fit into the focus-refined density map. Structural models of AP1 included its “hyper-unlocked” state (PDB ID: 4P6Z) (Jia et al., 2014), “unlocked” state (PDB ID: 4HMY) (Ren et al., 2013) and “locked” state (PDB ID: 1W63) (Heldwein et al., 2004). As the C-terminal μ-homology domain of δ-COP is prone to degradation, and also considering that this domain was omitted in the previous reconstruction of membrane-bound coatomer, we omitted this domain during the fitting. The appendage domains of β- and γ-COP were also omitted, as they are known to be linked flexibly to the trunk domains. After incorporating all these considerations, we found that the “hyper-open” conformation of F-subcomplex gave the best fit (Fig. 4D). Besides the densities occupied by the F-subcomplex, the unassigned density located underneath the “arch” on the β/γ-COP side could be assigned to the C-terminal μ-homology domain of δ-COP with good fit (Fig. 4D). As for the three structural models of AP1, only the “hyper-unlocked” conformation was found to give a reasonable fit (Fig. 4E). Furthermore, to test the possibility that the F-subcomplex can exist in multiple conformations, we performed 3D classification. However, after several cycles of classifications, we still could not detect any other conformation (Fig. S7).

Thus, the collective findings led us to conclude that the F-subcomplex exhibits an open conformation when coatomer is in its soluble form. Moreover, this conformation is similar to that seen previously for the membrane-bound form of coatomer (Dodonova et al., 2015). We further considered that the negative-stain EM approach provides a low-resolution structure of soluble coatomer. Thus, when also considering that flexible oscillation exists between the F-subcomplex and B-subcomplex, we did not attempt to join the two parts of structural reconstruction into one complete model of coatomer.

### CXMS analysis of coatomer in its cytosolic state

We next sought to further validate the structural reconstruction of coatomer we had generated above by taking another independent approach. Chemical cross-linking of proteins coupled with mass spectrometry (CXMS) has been developed in recent years as a way of validating structural models (Leitner et al., 2014; Yang et al., 2012). We found that there were 82 pairs of DSS or BS^3^ cross-linked lysine residues that could be detected with high confidence in the soluble form of coatomer, of which 50 were within the same domain and the remaining 32 occurred as cross-links between different domains. Among these inter-domain cross-links, 14 occurred within the subcomplexes of coatomer, while 18 occurred as links between the two subcomplexes (Table S2).

**Figure 5:**
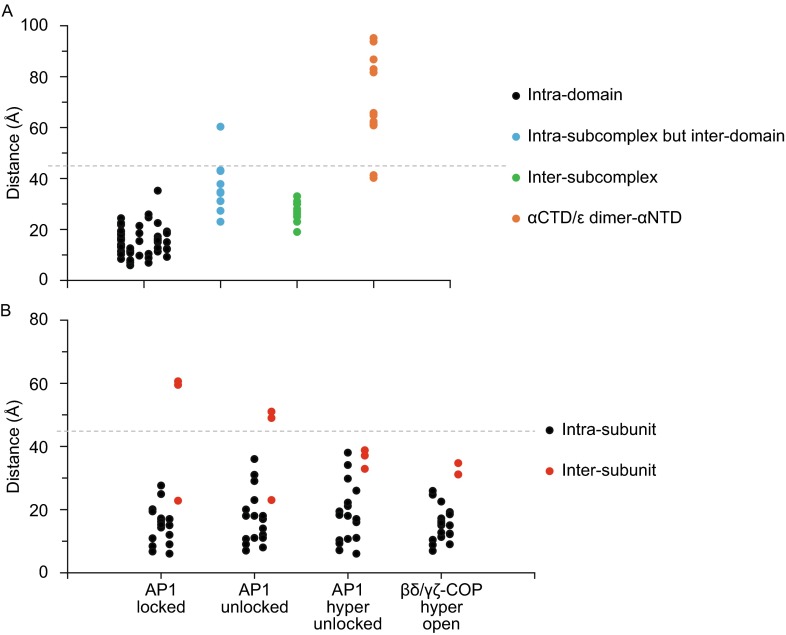
**Mapping CXMS cross-linked residue pairs onto structural models**. (A) The distances between Cα–Cα atoms of cross-linked residues pairs were measured based on the membrane-bound coatomer model (PDB ID: 5A1U), classified and plotted. (B) The distances between Cα–Cα atoms of cross-linked residues pairs within the adaptor F-subcomplex were measured based on four models as indicated, classified into intra-subunit (black) and inter-subunit (red) ones and plotted. The cut-off distance of 45 Å is represented by the dashed line

We next mapped all the CXMS pairs into the model of the previously elucidated membrane-bound coatomer and measured the Cα–Cα distances between the cross-linked residues (Table S2). The generally accepted Cα–Cα distance between two cross-linked lysine residues, when using DSS or BS^3^ as a cross-linker, is below ~35 Å (Politis et al., 2014). Since the model of the membrane-bound coatomer was generated from a 13-Å cryo-EM map (Dodonova et al., 2015), we relaxed the distance restriction of Cα–Cα to 45 Å. This resulted in all the intra-domain cross-links having Cα–Cα distances below 30 Å except for one pair that was below 35 Å (Table S2 and Fig. 5A). These findings suggested that the conformation of individual domains changes very little from the soluble to the membrane-bound form of coatomer.

As for the inter-domain cross-links, we found that 11 had distances greater than the cut-off value of 45 Å. Ten of these links were located between α^NTD^-COP and the α^CTD^/ε-COP heterodimer (Table S2 and Fig. 5A, orange). Furthermore, the cross-link distances between α^CTD^-COP and ε-COP were below the cut-off value (Table S2). Thus, these results suggested that the heterodimer of α^CTD^/ε-COP is structurally stable but changes its location between the soluble and membrane-bound forms of coatomer. In the soluble form, the heterodimer of α^CTD^/ε-COP interacts more closely with α^NTD^-COP. The 11th cross-link that was observed to have greater distance than the cut off value resides between the appendage domain of β-COP and the N-terminal domain of δ-COP (Table S2 and Fig. 5A). These two domains were far from each other in the membrane-bound form of coatomer, but were sufficiently close in the soluble form of coatomer to become cross-linked, thus suggesting a conformational change. Since the appendage domain of β-COP interacts with the N-terminal β-propeller domains of α-COP and contributes to the interaction between the two subcomplexes of coatomer in the membrane-bound state, we speculated that the conformational changes predicted by the crosslinking approach corresponds to the flexible oscillation between two subcomplexes of coatomer in its soluble form. Aside from the cross-links described above, all other inter-domain links were below the cut-off value of 45 Å. Thus, the CXMS approach also suggested that coatomer does not undergo a large conformational change when comparing its soluble versus membrane-bound form.

We also sought to validate our fitting results above on the F-subcomplex of coatomer by examining all 16 pairs of cross-linked residues within the main structure of the F-subcomplex. To do this, we removed from consideration the known flexible regions within this subcomplex, which are the appendage domains of β/γ-COP and the μ-homology domain of δ-COP. The three structural models of AP1 in different conformations were used as reference, as well as the membrane-bound form of coatomer. We found that all the Cα–Cα distances of the cross-links were below 35 Å for the “hyper-open” coatomer F-subcomplex and below 40 Å for the “hyper-unlocked” clathrin AP-1 (Fig. 5B and Table S3). However for the “unlocked” and the “locked” clathrin AP-1 models, there were some cross-links with larger distance than the cut-off value of 45 Å (Fig. 5B and Table S3). Thus, these results suggested that the F-subcomplex exhibits a relatively open conformation in the soluble form of coatomer. Notably, this conclusion is also consistent with the result above derived from the rigid-body fitting of the negative-stain EM map (Fig. 4D and 4E).

## Discussion

Using negative-stain EM and single-particle analysis, we have reconstructed the 3D structure of recombinant human coatomer in its soluble form (Fig. 4). This structure has been further validated by an independent approach using CXMS analysis. We then compared our reconstructed structure of soluble coatomer with a previously reconstructed structure of membrane-bound coatomer to reach a key conclusion, which is that soluble coatomer adopts largely a similar overall conformation as the membrane-bound form.

Coatomer is comprised of two subcomplexes. We find that the B-subcomplex forms an arch-like structure, which does not vary significantly when comparing the soluble versus the membrane-bound form of coatomer (Fig. 4C). The F-subcomplex also appears largely similar when comparing the two states of coatomer. Moreover, when compared with the previously elucidated structures of clathrin AP1, the F-subcomplex adopts a conformation that is similar to the open conformation of AP1. Thus, in contrast to the case of clathrin adaptors, for which membrane recruitment induces a large conformational change from “closed” to “open” conformation, coatomer remains in the “open” conformation regardless of whether it resides in the cytosol or on membrane (Fig. 4D).

We further note that flexibility in coatomer has been noted previously (Langer et al., 2008; Reinhard et al., 1999; Yip and Walz, 2011). Our current results reveal that flexibility can come from the two subcomplexes of coatomer having mobile interactions. We also acknowledge that flexibility can come domains within individual subunits of coatomer. These would include δ-COP, as well as portions of the α^CTD^/ε-COP heterodimer and the appendage domain of β/γ-COP. However, due to the limited resolution of the negative-stain EM approach, a more detailed assessment of these flexibilities will require future studies that can achieve a higher-resolution structure of coatomer.

**Figure 6:**
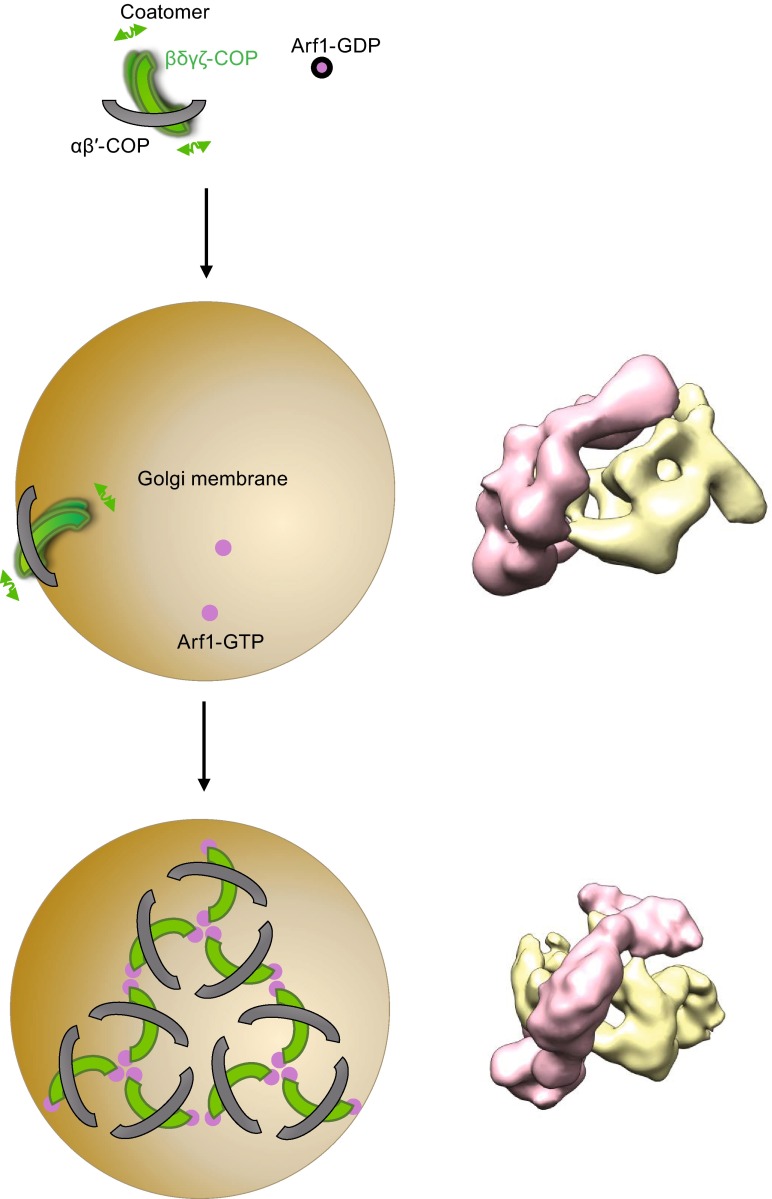
**A working model for the membrane recruitment of coatomers in promoting COPI vesicle formation**. The two subcomplexes (B-subcomplex in gray and F-subcomplex in green) of coatomer can oscillate in its cytosolic state. In the absence of ARF1-GTP (magenta), coatomer binds to membrane (brown) via the B-subcomplex, remodeling the membrane into tubules or “beads-on-string” morphology, for which the precise mechanism remains to be elucidated. Oscillation of the membrane-bound coatomer prevents its ability to drive vesicle formation. In the presence of membrane-anchored ARF1-GTP, the direct interaction between F-subcomplex and ARF1-GTP reduces the oscillation between the two subcomplexes, thereby driving the membrane-bound coatomer to a more stabilized (less oscillation) form. This stabilized coatomer is then able to assemble into oligomers, which together with ARF1, drives COPI vesicle formation

In any case, our current results suggest a speculative model for how coatomer is recruited onto membrane to participate in COPI vesicle formation (Fig. 6). Considering the flexible oscillation between the two subcomplexes of the coatomer, we propose that the interactions between membrane-anchored ARF1 and the F-subcomplex, together with the interaction between the scaffolding B-subcomplex and membrane, help to immobilize the interaction between the two subcomplexes of coatomer. This then triggers a conformational transition into the “active” form of coatomer on membrane. The subsequent self-assembly of coatomer, together with regions of ARF1 and coatomer interacting with the underlying membrane, induces curvature on this membrane, which eventually results in vesicle formation.

A new insight on coatomer has also come from our functional studies. Although early studies on COPI vesicle formation have used Golgi membrane, the use of simpler liposomal membranes in recent years has provided a more direct way of assessing how key factors, such as coatomer and ARF1, act to achieve vesicle formation (Bremser et al., 1999; Spang et al., 1998). In this regard, whereas ARF1 had been known to recruit coatomer from the cytosol to Golgi membrane in regulating the function of coatomer (Donaldson et al., 1992; Serafini et al., 1991a), the use of the simpler liposomal membrane has uncovered that ARF1 also possess a direct ability to induce membrane curvature (Beck et al., 2008; Krauss et al., 2008; Lundmark et al., 2008). In the current study, we have used of the simpler liposomal membrane to uncover that coatomer also possesses a direct ability to induce membrane curvature. To our knowledge, this behavior of coatomer has not been noted previously.

Studies on different coat complexes have revealed that single factors typically convert liposomes into tubules or “beads-on-string” morphology (Beck et al., 2008; Farsad et al., 2001; Lee et al., 2005a; Peter et al., 2004), while multiple factors are needed to convert liposomes into vesicles (Bremser et al., 1999; Matsuoka et al., 1998; Spang et al., 1998; Takei et al., 1998). We have observed similar effects, as the incubation of Golgi-like liposomes with either coatomer or ARF1 induces “beads-on-string” morphology, while their incubation together induces a more complete fission of liposomal membrane to generate COPI vesicles. These observations can be explained by the fission process requiring considerable force/strain being imposed on membrane. Thus, multiple factors acting in concert provide a way to generate such formidable force/strain.

At the mechanistic level, how protein factors induce membrane curvature is explained currently by two general mechanisms: i) introducing a wedge defect into one leaflet of the membrane bilayer that results in strain to promote curvature or ii) exerting a scaffolding mechanism that involves a curved protein structure binding to the underlying membrane through electrostatic interactions (McMahon and Gallop, 2005). The first mechanism explains how the ARF-like small GTPases induce membrane curvature, as they possess an N-terminal amphipathic helix that can insert into one leaflet of the membrane bilayer to induce membrane curvature (Beck et al., 2008; Bielli et al., 2005; Hariri et al., 2014; Lee et al., 2005a). How coatomer induces membrane curvature is less clear. Based on the recent structural reconstruction of coatomer on membrane, four sites in coatomer are predicted to contact the underlying membrane (Dodonova et al., 2015). Two of them are located in F-subcomplex and interact with the underlying membrane indirectly via ARF1. The other two are located in the B-subcomplex, and can potentially interact directly with membrane. However, because a high-resolution structure for these potential direct binding sites is currently unavailable, the precise mechanism by which coatomer induces membrane curvature remains to be determined in the future.

It is also notable that, besides ARF1, coatomer has been found to interact with multiple other proteins upon recruitment to membrane. These include cargo proteins (Cosson and Letourneur, 1994; Fiedler et al., 1996) and the catalytic regulators of ARF1, its GEF (Deng et al., 2009), and GAP (Goldberg, 1999; Lee et al., 2005b). Thus, an important future goal will be to pursue structural studies of these interactions, as they have the prospect of uncovering different ways by which the function of coatomer is regulated.

## Materials and methods

### Antibodies

For Western blotting, the rabbit polyclonal antibody specific for C-terminus of β-COP (H-300) was purchased from Santa Cruz Biotechnology. The anti-ARF1 antibody was raised against human ARF1(ΔN17) in rabbits.

### Molecular cloning, protein expression, and purification

The following cDNAs encoding for subunits of coatomer were synthesized commercially (Genewitz): α-COP isoform 1 (accession number NM_001098398.1), β-COP (accession NM_001144061.1), β′-COP (accession number NM_004766.2), γ-COP (accession number NM_016128.3), δ-COP isoform 1 (accession number NM_001655.4), ε-COP isofom 1 (accession number NM_007263.3), and ζ-COP isoform 1 (accession number NM_016057.2). The large plasmid containing all seven genes separated into two independent expression cassettes was developed by using ViperTEC method (to be published elsewhere) and transformed into DH10Bac competent *E. coli* for bacmid generation. The presence of all seven subunits of coatomer in the bacmid was verified by PCR before transfecting into Sf9 insect cells. The generation of baculovirus was performed by standard procedures. All Sf9 cells were cultured in serum free media (either Sf900-II, Life Technologies or ESF921, Expression Systems).

For protein expression, we infected 600 mL spinner flask cultures of Sf9 cell at a density of 2 × 10^6^ cells/mL with 5 mL of P2 virus. After 72 h, cells were harvested by centrifugation and suspended in cold lysis buffer (50 mmol/L Tris-Cl, pH 8.0, 100 mmol/L KCl, 10% glycerol, 1 mmol/L DTT) supplemented with protease inhibitors (*Cocktail*, complete-EDTA free, Roche Applied Science). Cells were lysed by ultra-sonication. After centrifugation, the cleared lysate was incubated with 1.5 mL pre-equilibrated Strep-Tactin Superflow beads (IBA). After washing with 50 mL lysis buffer, coatomer complex was eluted with 5 mL elution buffer (lysis buffer supplied with 5 mmol/L desthiobiotin). The elution was then loaded onto a 1 mL MonoQ anion exchange column (GE healthcare) pre-equilibrated with buffer A (20 mmol/L Bicine, pH 8.0, 100 mmol/L KCl, 10% glycerol, 1 mmol/L DTT) and eluted using a gradient from 100 mmol/L to 1 mol/L KCl with buffer B (20 mmol/L Bicine, pH 8.0, 1 mol/L KCl, 10% glycerol, 1 mmol/L DTT). Pooled fractions containing coatomer (approximately 0.35–0.4 mol/L KCl) were concentrated and then diluted in order for the final KCl concentration to be approximately 100 mmol/L. The two-step chromatography purification yielded above 90% pure coatomer at a protein concentration of ~10 mg/mL.

Recombinant human N-myristoylated Arf1 was purified from *E. coli* as previously described (Franco et al., 1996). Rat ArfGAP1 with a 10× His tag was expressed using BacMagic system and purified by affinity chromatography using Ni-NTA resin (GE Healthcare) and eluted by McAc300 buffer (50 mmol/L Tris-Cl, pH 8.0, 300 mmol/L KCl, 300 mmol/L imidazole).

### LC-MS protein identification

The gel band of interest was manually excised from native PAGE gel and then digested following standard procedures. The digested solution was then transferred to a sample vial for LC-MS analysis. LC-MS analysis was performed using a Thermo Finnigan LTQ linear ion trap mass spectrometer in line with a Thermo Finnigan Surveyor MS Pump Plus HPLC system. Peptides generated above were loaded onto a trap column (300SB-C18, 5 × 0.3 mm, 5 μm particle) (Agilent Technologies, Santa Clara CA) which was connected through a zero dead volume union to the self-packed analytical column (C18, 100 μm i.d × 100 mm, 3 μm particle) (SunChrom, Germany). The peptides were then eluted over a gradient (0%–45% B in 55 min, 45%–100% B in 10 min, where B = 80% Acetonitrile, 0.1% formic acid) at a flow rate of 500 nL/min and introduced online into the linear ion trap mass spectrometer (ThermoFisher Corporation, San Jose, CA) using nano electrospray ionization (ESI). Data dependent scanning was incorporated to select the 5 most abundant ions (one microscan per spectra; precursor isolation width 1.0 m/z, 35% collision energy, 30 ms ion activation, exclusion duration: 90 s; repeat count: 1) from a full-scan mass spectrum for fragmentation by collision induced dissociation (CID). MS data were analyzed using SEQUEST against NCBI human protein database; and results were filtered, sorted, and displayed using the Bioworks 3.2. Peptides with +1, +2, or +3 charge states were accepted if they were fully enzymatic and had a cross correlation (Xcorr) of 1.90, >2.5, and >3.0, respectively. The following residue modifications were allowed in the search: carbamidomethylation on cysteine and oxidation on methionine. Sequest was searched with a peptide tolerance of 3 Amu and a fragment ion tolerance of 1.0 Amu.

### Preparations of liposomes

Pure lipids were purchased from Avanti Polar lipids. Lipids were dissolved and mixed in either chloroform or methanol with composition similar to Golgi membrane (van Meer, 1998; van Meer et al., 2008): 45 mol% DOPC, 19 mol% DOPE, 5 mol% DOPS, 8 mol% PI, 7 mol% SM, 16 mol% cholesterol. After evaporation, the dry lipid film was suspended and hydrated in Assay buffer (25 mmol/L HEPES, pH 7.2, 25 mmol/L KCl, 2.5 mmol/L Mg(OAc)_2_). After vortexing and repeated freezing/thawing, liposomes were further extruded through polycarbonate filter with 0.2 μm pore.

### Membrane binding and vesiculation assays

Incubation was carried out in 50 μL AS buffer (25 mmol/L Hepes, pH 7.2, 25 mmol/L KCl, 0.2 mol/L sucrose, 2.5 mmol/L Mg(OAc)_2_) for 30 min at 37°C with myr-Arf1 (4.8 μmol/L), coatomer (182 nmol/L), GDP (1 mmol/L)/GTPγS (25 μmol/L), and liposome (0.2 mg/mL). EDTA (5 mmol/L) was added firstly for guanine nucleotides exchange of Arf1, and then Mg(OAc)_2_ (10 mmol/L) was supplied followed by the addition of other proteins into the incubation reactions. For studies of ArfGAP1, these incubation was carried out for 25 min at 37°C followed by additional incubation with ArfGAP1 (1 μmol/L) for 5 min at 37°C. After incubation, 10 μL of the reaction mix was reserved for negative staining and EM observation, and the remaining 40 μL reaction mix was ultracentrifuged (250,000 ×*g*, 30 min, 4°C) through 500 μL AS buffer to pellet membranes. After resuspending with AS buffer, the membranes and their bound proteins were analyzed by SDS-PAGE and Western blotting.

For purification of COPI-coated vesicles, the incubation volume was scaled up to 250 μL with liposome at a concentration of 1.5 mg/mL. After incubation, the reconstituted COPI vesicles were isolated from membranes and soluble proteins by subsequent centrifugation through two sucrose cushions in AS buffer (37.5%, 50 μL; 45%, 10 μL), essentially as previously described (Beck et al., 2009). After centrifugation for 50 min at 100,000×*g*, the fraction at the interface of 37.5%/45% sucrose cushion was collected and equally divided into two aliquots for either Western blotting or negative stain EM analysis.

### Negative stain electron microscopy

For soluble coatomer, holy carbon grids coated with thin (5 nm) continuous carbon films, which was manufactured in-house, were used. Samples were applied to the glow-discharged grids and adsorbed for 1 min at RT. After washing with buffer, the grids were stained with 2% (*w*/*v*) uranyl acetate for 1 min at RT. For liposomes and vesicles, the incubation reaction solutions were directly applied onto glow-discharged EM grids (LifeTrust) coated with continuous carbon film and stained with 2% (*w*/*v*) uranyl acetate for 1 min at RT.

Images were collected on a 200 kV FEI F200C Talos field emission transmission electron microscopy equipped with a 4 k × 4 K Ceta charge-coupled device camera (FEI, Netherlands). Single particle data were collected automatically with SerialEM software package (Mastronarde, 2005) at the magnification of 57,000 with a pixel size of 1.81 Å.

### Electron tomography and sub-tomogram averaging

The same grid from the single particle data collection was applied for tilt series tomography data collection. Data were collected using SerialEM software package (Mastronarde, 2005) at the magnification of 57,000 with a pixel size of 1.81 Å. The angular range was from −55° to +55° with an increment of 5° and the total electron dose was 80–100 e/Å^2^. Processing was performed on binned data with a pixel size of 3.6 Å. Gctf (Zhang, 2016) was used to estimate the defocus, which ranges from 5.0 μm to 8.0 μm. The tilt series were aligned using MarkerAuto (Han et al., 2015) before reconstruction using IMOD (Kremer et al., 1996). 632 sub-volumes with a box size of 84 pixels were picked using IMOD (Kremer et al., 1996) and extracted using RELION1.4 (Bharat et al., 2015). Subsequently, sub-volume alignment and refinement were performed using RELION1.4 without CTF model. The initial model used in RELION1.4 refinement was a random rotational average of all the extracted sub-volumes (Bharat et al., 2015). The reconstruction after refinement was used as an initial model for the further single particle data processing.

### Single particle analysis

Micrographs were screened out due to severe astigmatism, drift, inappropriate defocus, contamination, particle aggregation, and imperfect staining. Particles were first manually picked from 20 micrographs and class averaged using RELION1.3 (Scheres, 2012). Next, the class average results were used for template-based particle autopicking by Gautomatch (by Kai Zhang, MRC). To limit model bias, we applied a 40 Å low-pass filter to these templates. An initial dataset of 353,777 particles was submitted to multiple cycles of reference-free 2D classification in RELION1.3 to exclude bad particles, such as contamination or overlapping particles. The 109,342 selected particles were used for 3D reconstruction with the initial model that was obtained from sub-tomogram average and filtered to 60 Å. After several cycles of 3D classification and refinement, a 2D classification step using the alignment parameters from the 3D refinement was performed to check the alignment accuracy and to further exclude particles liable to misalignment. The resulting 60,007 qualified particles were used for final 3D refinement and the subsequent “focus” refinement (Bai et al., 2015). For “focus” refinement, we segmented the final reconstruction map using the “Segmentation” tool in UCSF Chimera (Pettersen et al., 2004). The segmented densities were grouped into two sets, which were assigned as the two subcomplexes according to the rigid-body fitting of the membrane-bound coatomer structure. Then, the grouped densities were further extracted to a new volume data, and used for subtraction of the corresponding signals from the raw particles images using “relion_project” commands. The resulting new particle stacks were used as input in the subsequent 3D auto refine and classification processes.

### CXMS

CXMS analysis was performed essentially as described previously (Ding et al., 2016). Briefly, in each 20 μL cross-linking reaction, about 12 μg of the purified COPI complex was incubated with BS^3^, DSS, or Sulfo-GMBS at a molar ratio of 1:8 or 1:4 (protein : cross-linker) for one hour at room temperature. The reactions were quenched with 20 mmol/L NH_4_HCO_3_ for BS^3^ and DSS reactions, or with 20 mmol/L DTT, 20 mmol/L NH_4_HCO_3_ for Sulfo-GMBS cross-linking. Proteins were precipitated with ice-cold acetone, resuspended in 8 mol/L urea, 100 mmol/L Tris, pH 8.5. After Trypsin digestion, the LC-MS/MS analysis was performed on an Easy-nLC 1000 UPLC (Thermo Fisher Scientific) coupled to a Q Exactive-Orbitrap mass spectrometer (Thermo Fisher Scientific). Peptides were loaded onto a pre-column (75 μm ID, 8 cm long, packed with ODS-AQ 12 nm–10 μm beads from YMC Co., Ltd.) and separated on an analytical column (75 μm ID, 11 cm long, packed with Luna C18 1.8 μm 100 Å resin from Welch Materials) using an acetonitrile gradient from 0%–21% in 83 min at a flow rate of 200 nL/min. The top 15 most intense precursor ions from each full scan (resolution = 70,000) were isolated for HCD MS2 (resolution = 17,500; normalized collisional energy = 27) with a dynamic exclusion time of 20 s. Precursors with 1+, 2+, 7+ or above, or unassigned charge states were excluded. Cross-linked peptides were identified using pLink (Yang et al., 2012) with a 5% FDR cutoff at the spectral level and then an *E*-value cutoff at 0.001.

The Cα–Cα distance between two cross-linked residues were measured in PyMOL (The PyMOL Molecular Graphics System, Version 1.74, Schrodinger, LLC.). For the three structural models of clathrin AP-1 homologues, we first built models with human coatomer sequence, taking the crystal structure models (PDB ID: 4P6Z/4HMY/1W63) as reference using *Modeller* software (Šali et al., 1995), and then the Cα–Cα distance between cross-linked residues were measured in PyMOL.

## Electronic supplementary material

Below is the link to the electronic supplementary material.

Supplementary material 1 (PDF 276 kb)

Supplementary material 2 (PDF 1167 kb)
