# Supplementary material for: Structural characterization of coatomer in its cytosolic state
Source: Protein Cell. 2016 Jul 29;7(8):586–600. doi: 10.1007/s13238-016-0296-z (PMC4980336; doi:10.1007/s13238-016-0296-z)
Supplement: Supplementary file 1 — Supplementary material 1 (PDF 276 kb) [file 13238_2016_296_MOESM1_ESM.pdf]

SUPPLEMENTAL TEXT

Effect of membrane cholesterol COPI vesicle formation

A previous study had found cholesterol to be relatively depleted from COPI vesicles reconstituted from Golgi membrane (Brugger et al., 2000). Thus, we also examined how cholesterol affects vesicle formation using liposomal membrane. The molar ratios of cholesterol to total phospholipids are 1:10 for ER membrane, 1:5 for Golgi membrane, 1:2 for endosome membrane and 1:1 for plasma membrane. We thus generated liposomes at various relative cholesterol levels (0:1, 1:10, 1:5, 1:2 and 1:1 respectively) and used these liposomes for vesiculation assay and the subsequent EM observations. For each vesiculation reaction, we performed three independent experiments and prepared three EM grids. And for each grid, we randomly selected five squares and recorded 18 micrographs for each square. During image processing, all the micrographs were merged into one dataset to perform particle picking and reference-free 2D classification. After that, the vesicle numbers per micrographs and the corresponding micrograph names were extracted for further statistical analysis

We investigated the number of vesicles per micrograph, and found that there was a dramatic decrease of vesiculation efficiency when the cholesterol level increased above 1:5 (**Figure S4A**), which is the cholesterol ratio predicted for Golgi membrane (van Meer et al., 2008). Further analysis showed that the size of vesicles was also affected, with diameters becoming larger with increase in cholesterol concentration (**Figure S4B**). Notably, these findings are consistent with a previous finding that COPI vesicles reconstituted from Golgi membrane have relatively depleted level of cholesterol (Brugger et al., 2000). As such, our cholesterol data supports the use of liposomal membrane to assay for COPI vesicle formation as a way of testing the functionality of the recombinant coatomer that we have generated for the current study.

SUPPLEMENTAL METHODS

Preparations of liposomes

Pure lipids were purchased from Avanti Polar lipids. Lipids were dissolved and mixed in either chloroform or methanol with cholesterol-gradient composition. The molar ratio of cholesterol to total phospholipids was set to 0:1, 1:10, 1:5, 1:2 and 1:1, which can also be represented by molar ratio of cholesterol to total lipids: 0 mol% (ratio: 0:1), 8 mol% (ratio: 1:10), 16 mol% (ratio: 1:5), 42 mol% (ratio: 1:2) and 83 mol% (ratio: 1:1). For phospholipids (DOPC/DOPE/DOPS/DOPI/SM), their relative proportions were set to be similar to that of Golgi membrane (van Meer, 1998; van Meer et al., 2008). After evaporation, the dry lipid film was suspended and hydrated in Assay buffer (25 mM Hepes, pH 7.2, 25 mM KCl, 2.5 mM $Mg(OAc)_2$). After vortexing and repeated freezing/thawing, liposomes were further extruded through polycarbonate filter with 0.2 μm pore.

In vitro membrane binding and vesiculation assay

Incubation was carried out in 50 μl AS buffer (25 mM Hepes, pH 7.2, 25 mM KCl, 0.2 M sucrose, 2.5 mM $Mg(OAc)_2$) for 30 min at 37°C with myr-Arf1 (4.8 μM), coatomer (182 nM), GTPγS (25 μM) and liposome (0.2 mg/ml).

Negative stain electron microscopy

After incubation, the reaction solutions were directly applied onto glow-discharged EM grids (LifeTrust) coated with continuous carbon film and stained with 2% (w/v) uranyl acetate for 1 min at RT.

Images were collected on a 200kV FEI F200C Talos field emission transmission electron microscope equipped with a 4k×4K Ceta charge-coupled device camera (FEI, Netherlands) at magnification of 57,000 fold with a pixel size of 1.81 Å.

SUPPLEMENTAL FIGURE LEGEND

Figure S1. Coatomer purification and subunit detection. The subunits with different tags are indicated accordingly.

Figure S2. **SDS-PAGE of purified myristoylated Arf1 and ArfGAP1**. (**A**) Coomassie-blue stained SDS-PAGE of myristoylated Arf1 eluted from Mono S chromatography. The fractions indicated, which contains only myristoylated Arf1, were pooled for further functional assays. (**B**) Coomassie-blue stained SDS-PAGE of ArfGAP1 eluted from Strep II affinity chromatography.

Figure S3. Characterizing vesiculated liposomes as COPI vesicles. (**A**) Negative-stain EM image of the purified vesicles from sucrose density sedimentation. Two vesicles are selected and magnified in the insets. The white scale-bar for the whole image is 500 nm. The black scale-bar in the inset is 100 nm. (**B**) Western blotting analysis of the purified vesicles.

Figure S4. Effect of cholesterol on COPI vesicle formation. (**A**) Statistical histogram of vesicle number vs. different concentrations of cholesterol. All error bars represent s.d. from three independent vesicle reconstitution experiments. For each experiment, N = 90. The statistical significances of differences are expressed as ** for $p < 0.01$ and * for $p < 0.05$. (**B**) Statistical histograms of the vesicle number vs. vesicle diameters for different concentrations of cholesterol. All error bars represent s.d. from three independent vesicle reconstitution experiments. For each experiment, N = 90.

Figure S5. Negative-stain EM analysis of the recombinant human coatomer. (**A**) Raw image of negatively stained coatomer with the individual particles circled; scale bar, 50 nm. (**B**) 2D class averages of the negatively stained coatomer, box size = 30 nm. (**C**) Representative 2D class averages showing the two major parts of coatomer described in the text with some flexible extra densities extending outside these two parts as indicated by the schematic below. Arrows point to potential dynamic parts of coatomer.

Figure S6. 3D reconstruction of the recombinant human coatomer. (**A**) “Focus” refinement process (see also **MATERIALS AND METHODS**). The iso-surface (displayed at levels corresponding to the volume of a single coatomer complex or as separated subcomplexes) and the ortho-slices are both displayed. (**B**) FSC curves of 3D reconstructions.

Figure S7. “Focus” classification of the adaptor F-subcomplex. The iso-surface (displayed at the same level corresponding to the volume of the F-subcomplex) and the ortho-slices are both displayed.

Table S1. Mass spectrometry analysis for identifying the degraded fragment of δ-COP.

Start	End	Observed	Mr(expt)	Mr(calc)	ppm	Peptide
1	14	1433.0720	1432.0647	1431.7942	189	-.MVLLAAAVCTKAGK.A
20	26	910.6010	909.5937	909.4378	171	R.QFVEMTR.T
27	38	1316.0470	1315.0397	1314.7659	208	R.TRIEGLLAAFPK.L
39	55	1977.3640	1976.3567	1975.9786	191	K.LMNTGKQHTFVETESVR.Y
39	55	1993.3470	1992.3397	1991.9735	184	K.LMNTGKQHTFVETESVR.Y + Oxidation (M)
45	55	1332.9480	1331.9407	1331.6470	221	K.QHTFVETESVR.Y
56	63	1057.7290	1056.7217	1056.4950	215	R.YVYQPMEK.L
64	72	1081.7970	1080.7897	1080.6253	152	K.LYMVLITTK.N
73	84	1417.0550	1416.0477	1415.7256	228	K.NSNILEDLETLR.L
89	95	936.6520	935.6447	935.4535	204	R.VIPEYCR.A
132	145	1756.1550	1755.1477	1754.7934	202	R.TFTEMDSHEEKVFR.A
132	145	1772.1530	1771.1457	1770.7883	202	R.TFTEMDSHEEKVFR.A + Oxidation (M)
211	223	1234.9910	1233.9837	1233.6830	244	K.VAPAPARPSGPSK.A
287	307	2384.3280	2383.3207	2383.0970	93.9	R.DGGLQNMELHGMIMLHISDEK.F + Oxidation (M)

Table S2. Identified cross-linked residues and their Ca-Ca distance measured in the membrane-bound coatomer model (Distances greater than the cut-off value of 45 Å are colored in red).

No.	Subunit1	AA	Domain 1	Subunit2	AA	Domain2	ID Score	Distance
	**Intra-domain**							
1	α	8	1st β-propeller	α	46	1st β-propeller	156	14
2	α	198	1st β-propeller	α	238	1st β-propeller	53	13
3	α	446	2nd β-propeller	α	480	2nd β-propeller	341	19
4	α	411	2nd β-propeller	α	441	2nd β-propeller	235	14
5	α	411	2nd β-propeller	α	438	2nd β-propeller	109	14
6	α	350	2nd β-propeller	α	398	2nd β-propeller	95	24
7	α	337	2nd β-propeller	α	362	2nd β-propeller	28	8
8	α	707	α-solenoid	α	727	α-solenoid	280	18
9	α	680	α-solenoid	α	707	α-solenoid	86	17
10	α	607	α-solenoid	α	616	α-solenoid	86	16
11	α	730	α-solenoid	α	736	α-solenoid	82	11
12	α	707	α-solenoid	α	736	α-solenoid	64	12
13	α	707	α-solenoid	α	724	α-solenoid	22	16
14	α	707	α-solenoid	α	730	α-solenoid	15	13
15	α	1123	CTD	α	1152	CTD	180	10
16	α	1084	CTD	α	1123	CTD	174	18
17	α	1002	CTD	α	1189	CTD	35	22
18	α	1080	CTD	α	1123	CTD	28	23
19	α	1081	CTD	α	1123	CTD	22	19
20	β‘	8	1st β-propeller	β‘	10	1st β-propeller	94	6
21	β‘	69	1st β-propeller	β‘	131	1st β-propeller	34	12
22	β‘	386	2nd β-propeller	β‘	419	2nd β-propeller	568	12
23	β‘	328	2nd β-propeller	β‘	346	2nd β-propeller	342	7
24	β‘	313	2nd β-propeller	β‘	346	2nd β-propeller	89	8
25	β‘	313	2nd β-propeller	β‘	569	2nd β-propeller	18	11
26	β‘	771	α-solenoid	β‘	779	α-solenoid	182	13
27	ε	42	1st β-propeller	ε	74	1st β-propeller	320	21
28	ε	278	1st β-propeller	ε	288	1st β-propeller	319	10
29	ε	158	1st β-propeller	ε	288	1st β-propeller	35	19
30	ε	278	1st β-propeller	ε	293	1st β-propeller	19	15
31	β	36	trunk	β	43	trunk	72	10
32	β	501	trunk	β	535	trunk	68	25

33	β	361	trunk	β	366	trunk	62	9
34	β	501	trunk	β	534	trunk	32	26
35	β	32	trunk	β	37	trunk	31	7
36	γ	514	trunk	γ	538	trunk	49	17
37	γ	465	trunk	γ	540	trunk	31	23
38	γ	145	trunk	γ	175	trunk	25	11
39	γ	175	trunk	γ	213	trunk	24	15
40	γ	262	trunk	γ	300	trunk	21	13
41	γ	205	trunk	γ	238	trunk	17	16
42	γ	238	trunk	γ	313	trunk	17	16
43	γ	604	appendage	γ	818	appendage	36	35
44	δ	14	NTD	δ	38	NTD	37	9
45	δ	335	μ homology	δ	363	μ homology	214	15
46	δ	335	μ homology	δ	347	μ homology	199	12
47	δ	322	μ homology	δ	335	μ homology	118	19
48	δ	281	μ homology	δ	363	μ homology	111	12
49	δ	322	μ homology	δ	476	μ homology	102	19
50	δ	448	μ homology	δ	476	μ homology	26	19
	**Inter-domains but Intra-subunit**							
51	α	4	1st β-propeller	α	540	2nd β-propeller	147	38
52	α	583	2nd β-propeller	α	607	α-solenoid	144	43
53	α	583	2nd β-propeller	α	616	α-solenoid	16	34
54	α	238	1st β-propeller	α	1189	CTD	45	95
55	α	411	2nd β-propeller	α	1002	CTD	31	41
56	α	411	2nd β-propeller	α	1189	CTD	30	61
57	α	480	2nd β-propeller	α	1189	CTD	30	65
58	α	583	2nd β-propeller	α	997	CTD	21	62
59	α	526	2nd β-propeller	α	997	CTD	21	61
60	α	583	2nd β-propeller	α	1189	CTD	20	83
61	α	526	2nd β-propeller	α	1189	CTD	15	82
62	β'	10	1st β-propeller	β'	318	2nd β-propeller	17	27
63	β'	336	2nd β-propeller	β'	615	α-solenoid	15	23
64	β	378	trunk	β	932	appendage	54	43
	**Inter-domains and Inter-subunits**							
65	α	46	1st β-propeller	β	771	appendage	120	30
66	α	81	1st β-propeller	β	771	appendage	33	19
67	α	487	2nd β-propeller	β	949	appendage	67	28
68	α	526	2nd β-propeller	β	949	appendage	42	26
69	α	490	2nd β-propeller	β	949	trunk	38	23
70	α	46	1st β-propeller	δ	14	N-terminal	99	27

71	α	46	1st β-propeller	δ	38	N-terminal	32	33
72	β'	336	2nd β-propeller	β	362	trunk	33	25
73	β'	615	α-solenoid	β	361	trunk	28	30
74	β'	615	α-solenoid	β	362	trunk	47	31
75	β	932	appendage	δ	44	N-terminal	19	60
76	β	534	trunk	ζ	39	core	15	31
77	β	535	trunk	ζ	39	core	17	35
78	γ	313	trunk	ζ	51	core	46	31
79	α	238	1st β-propeller	ε	42	core	21	87
80	α	238	1st β-propeller	ε	278	core	17	94
81	α	350	2nd β-propeller	ε	42	core	16	66
82	α	1002	C-terminal	ε	167	core	77	40

Table S3. Identified cross-linked residues within F-subcomplex and their Ca-Ca distance measured in four structural models with different conformations (Distances greater than the cut-off value of 45 Å are colored in red).

No.	Subunit 1	AA	Domain 1	Subunit 2	AA	Domain2	models for distance measure			
							AP-1 locked	AP-1 unlocked	AP-1 hyper unlocked	coatomer hyper open
	**Intra-subunit**									
1	β	36	trunk	β	43	trunk	11	11	10	10
2	β	501	trunk	β	535	trunk	20	20	19	25
3	β	361	trunk	β	366	trunk	8	9	9	9
4	β	501	trunk	β	534	trunk	19	18	18	26
5	β	32	trunk	β	37	trunk	7	7	7	7
6	γ	514	trunk	γ	538	trunk	25	31	30	17
7	γ	465	trunk	γ	540	trunk	28	29	21	23
8	γ	145	trunk	γ	175	trunk	14	11	11	11
9	γ	175	trunk	γ	213	trunk	17	18	18	15
10	γ	262	trunk	γ	300	trunk	17	23	22	13
11	γ	205	trunk	γ	238	trunk	16	36	38	16
12	γ	238	trunk	γ	313	trunk	15	31	34	16
13	γ	604	appendage	γ	818	appendage	/	/	/	35
14	δ	14	NTD	δ	38	NTD	17	17	16	9
15	δ	335	μ homology	δ	363	μ homology	6	14	6	15
16	δ	335	μ homology	δ	347	μ homology	17	12	11	12
17	δ	322	μ homology	δ	335	μ homology	15	17	17	19
18	δ	281	μ homology	δ	363	μ homology	12	11	11	12
19	δ	322	μ homology	δ	476	μ homology	9	8	26	19
20	δ	448	μ homology	δ	476	μ homology	17	18	17	19
	**Inter-subunit**									
21	β	534	trunk	ζ	39	core	60	49	37	31
22	β	535	trunk	ζ	39	core	61	51	39	35
23	γ	313	trunk	ζ	51	core	23	23	33	31
24	β	932	appendage	δ	44	N-terminal	/	/	/	60
