# Supplementary material for: Structural characterization of coatomer in its cytosolic state
Source: Protein Cell. 2016 Jul 29;7(8):586–600. doi: 10.1007/s13238-016-0296-z (PMC4980336; doi:10.1007/s13238-016-0296-z)
Supplement: Supplementary file 2 — Supplementary material 2 (PDF 1167 kb) [file 13238_2016_296_MOESM2_ESM.pdf]

Figure S1

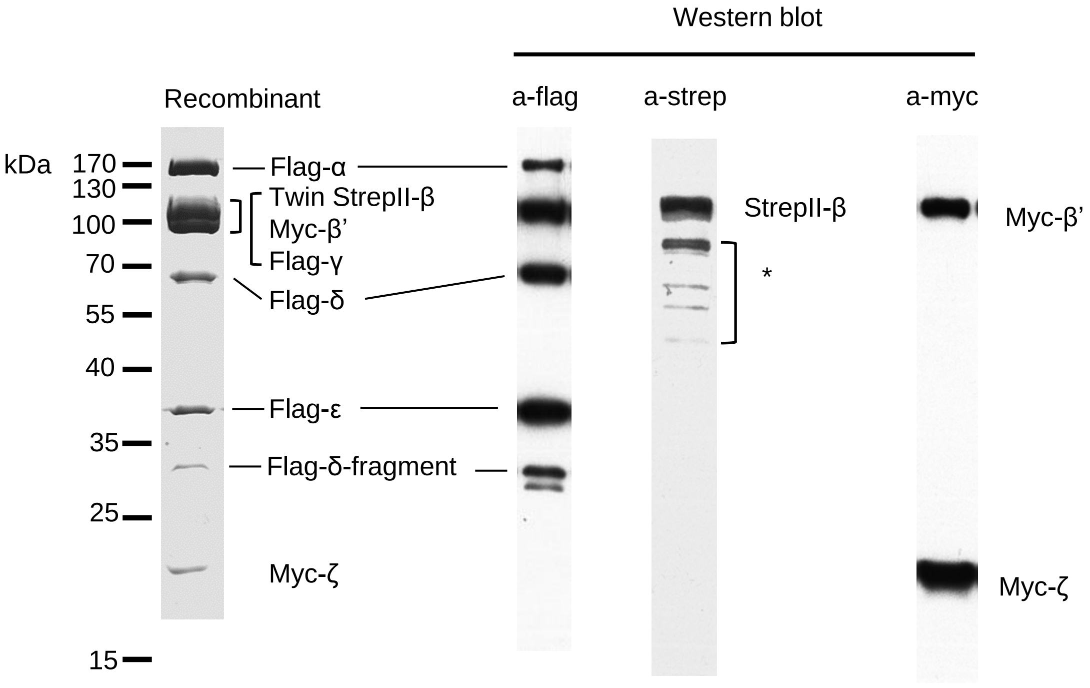

Figure S1. Coatomer purification and subunit detection. The subunits with different tags are indicated accordingly.

Figure S2

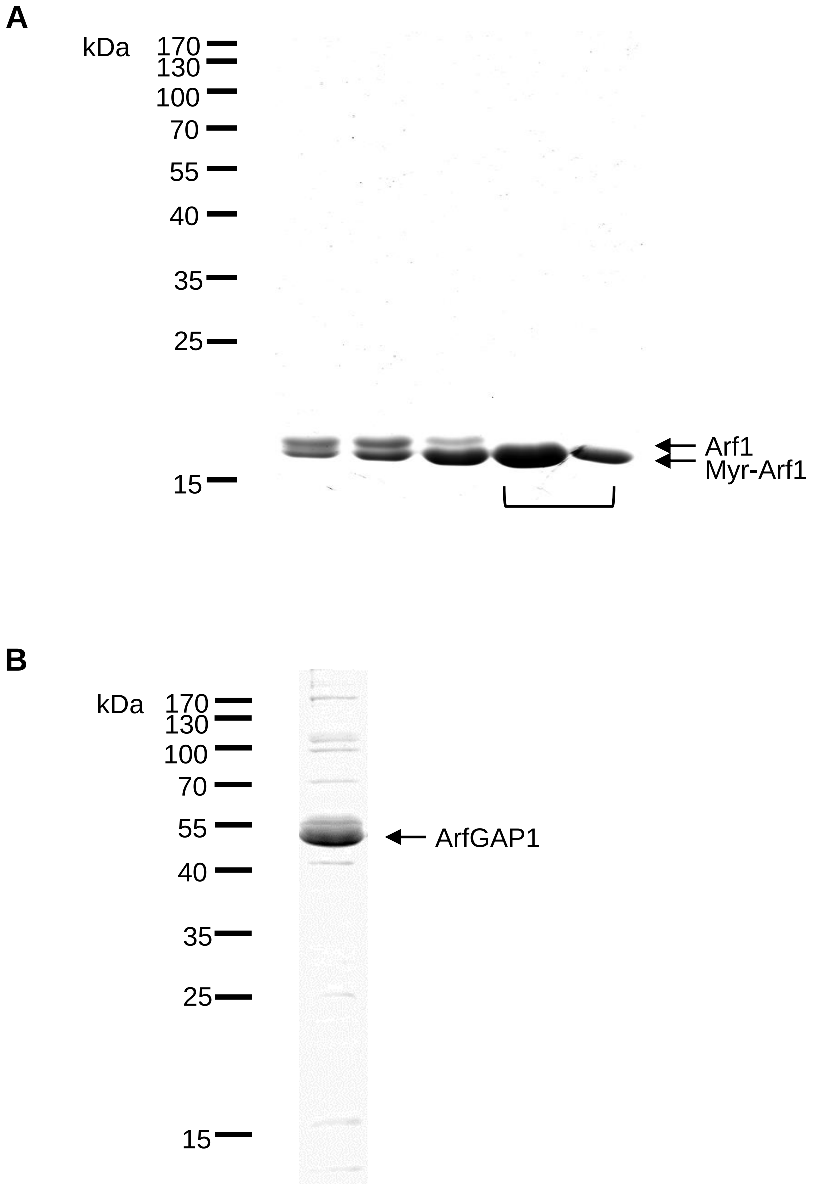

Figure S2. **SDS-PAGE of purified myristoylated Arf1 and ArfGAP1**. (**A**) Coomassie-blue stained SDS-PAGE of myristoylated Arf1 eluted from Mono S chromatography. The fractions indicated, which contains only myristoylated Arf1, were pooled for further functional assays. (**B**) Coomassie-blue stained SDS-PAGE of ArfGAP1 eluted from Strep II affinity chromatography.

Figure S3

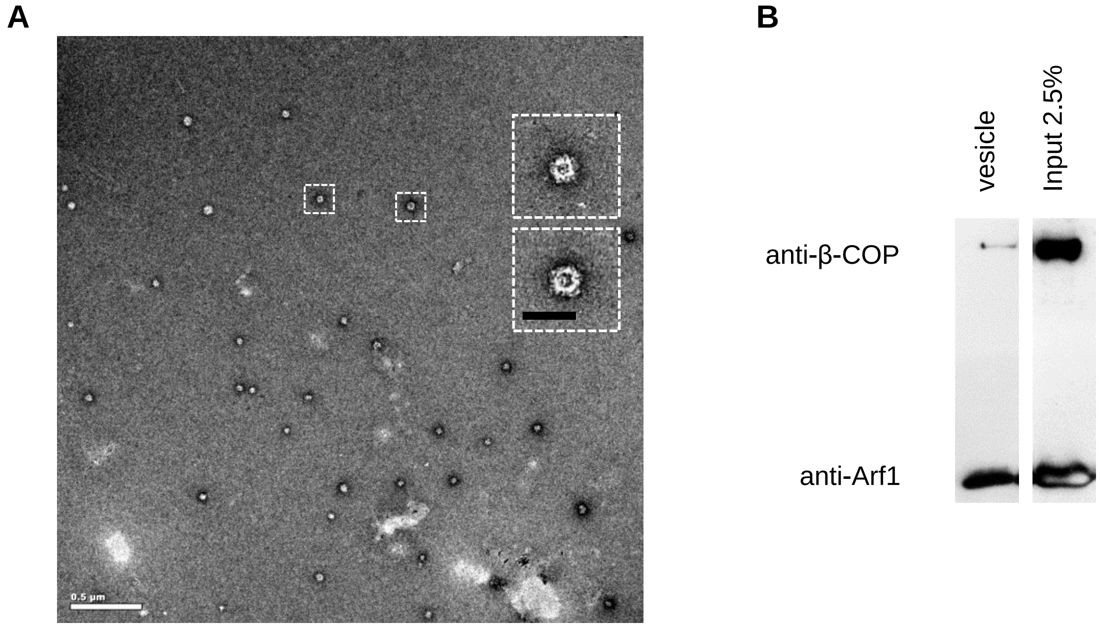

Figure S3. Characterizing vesiculated liposomes as COPI vesicles. (**A**) Negative-stain EM image of the purified vesicles from sucrose density sedimentation. Two vesicles are selected and magnified in the insets. The white scale-bar for the whole image is 500 nm. The black scale-bar in the inset is 100 nm. (**B**) Western blotting analysis of the purified vesicles.

Figure S4

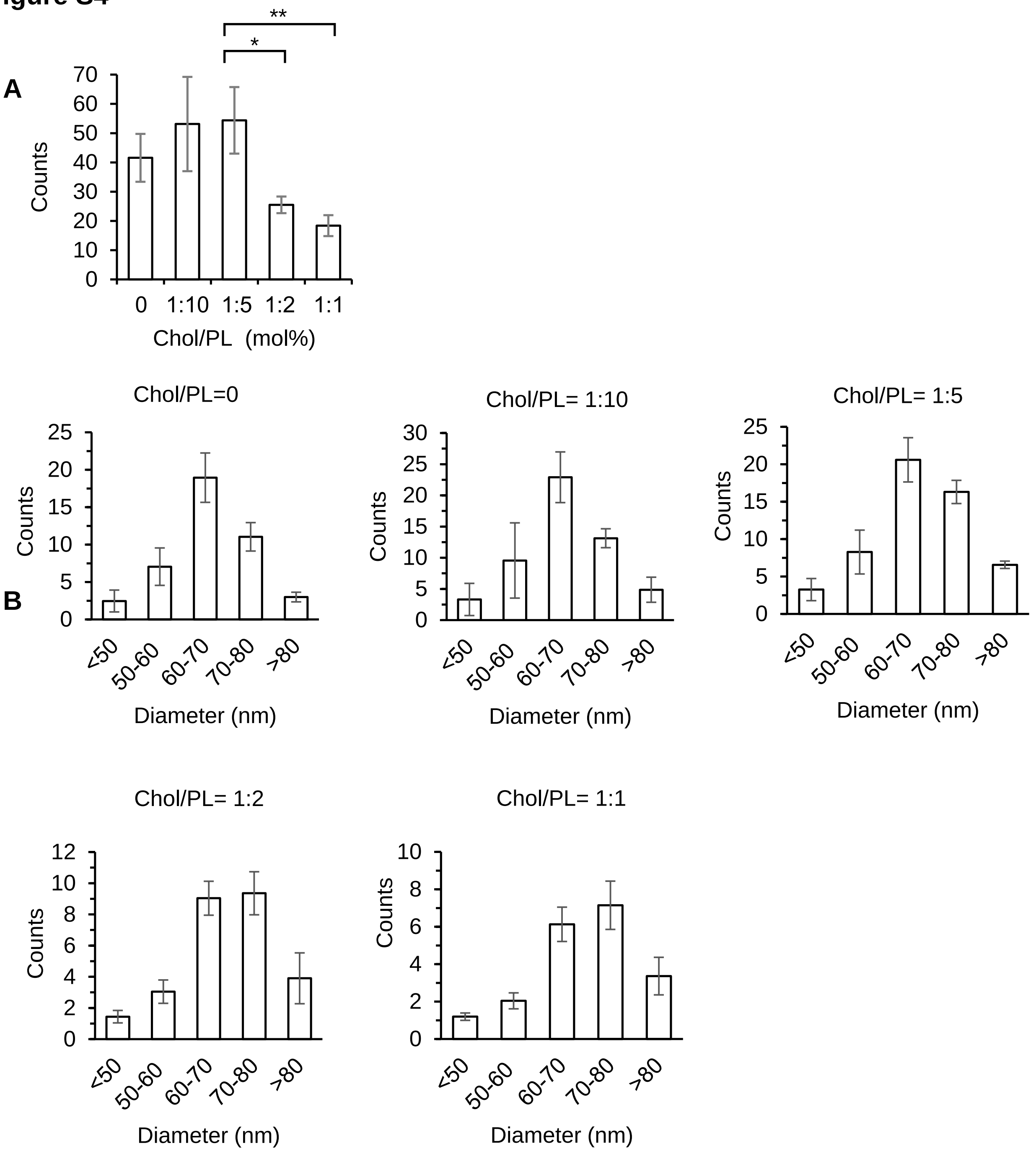

Figure S4. Effect of cholesterol on COPI vesicle formation. (**A**) Statistical histogram of vesicle number vs. different concentrations of cholesterol. All error bars represent s.d. from three independent vesicle reconstitution experiments. For each experiment, N = 90. The statistical significances of differences are expressed as ** for $p < 0.01$ and * for $p < 0.05$. (**B**) Statistical histograms of the vesicle number vs. vesicle diameters for different concentrations of cholesterol. All error bars represent s.d. from three independent vesicle reconstitution experiments. For each experiment, N = 90.

Figure S5

A

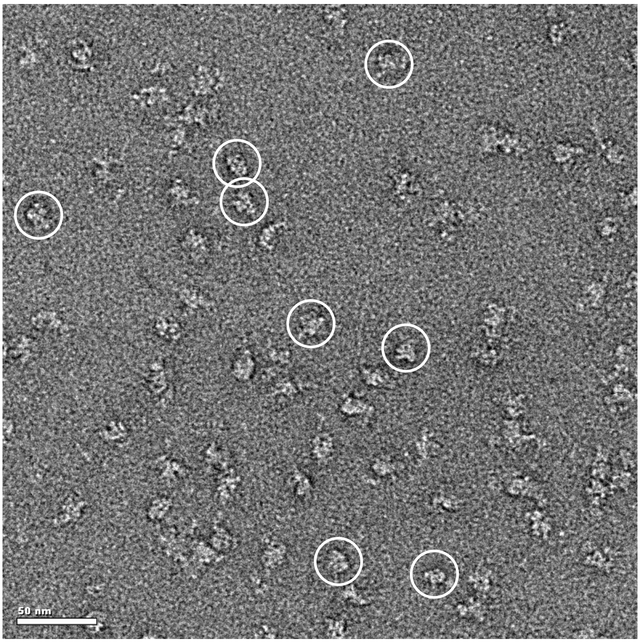

C

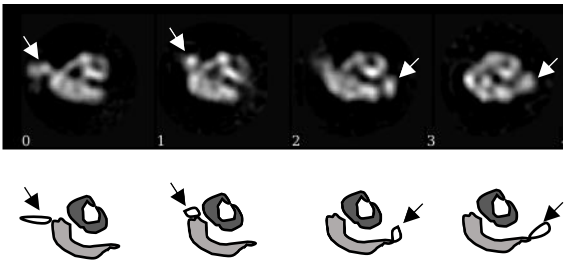

B

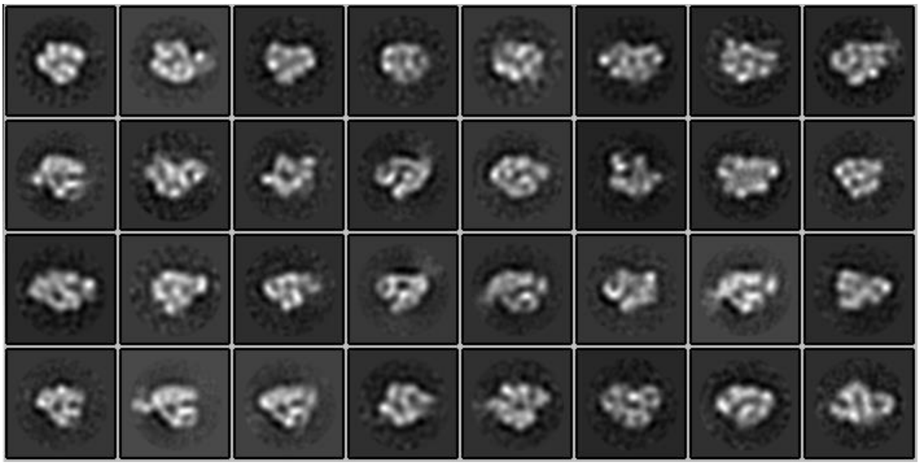

Figure S5. Negative-stain EM analysis of the recombinant human coatomer. (**A**) Raw image of negatively stained coatomer with the individual particles circled; scale bar, 50 nm. (**B**) 2D class averages of the negatively stained coatomer, box size = 30 nm. (**C**) Representative 2D class averages showing the two major parts of coatomer described in the text with some flexible extra densities extending outside these two parts as indicated by the schematic below. Arrows point to potential dynamic parts of coatomer.

Figure S6

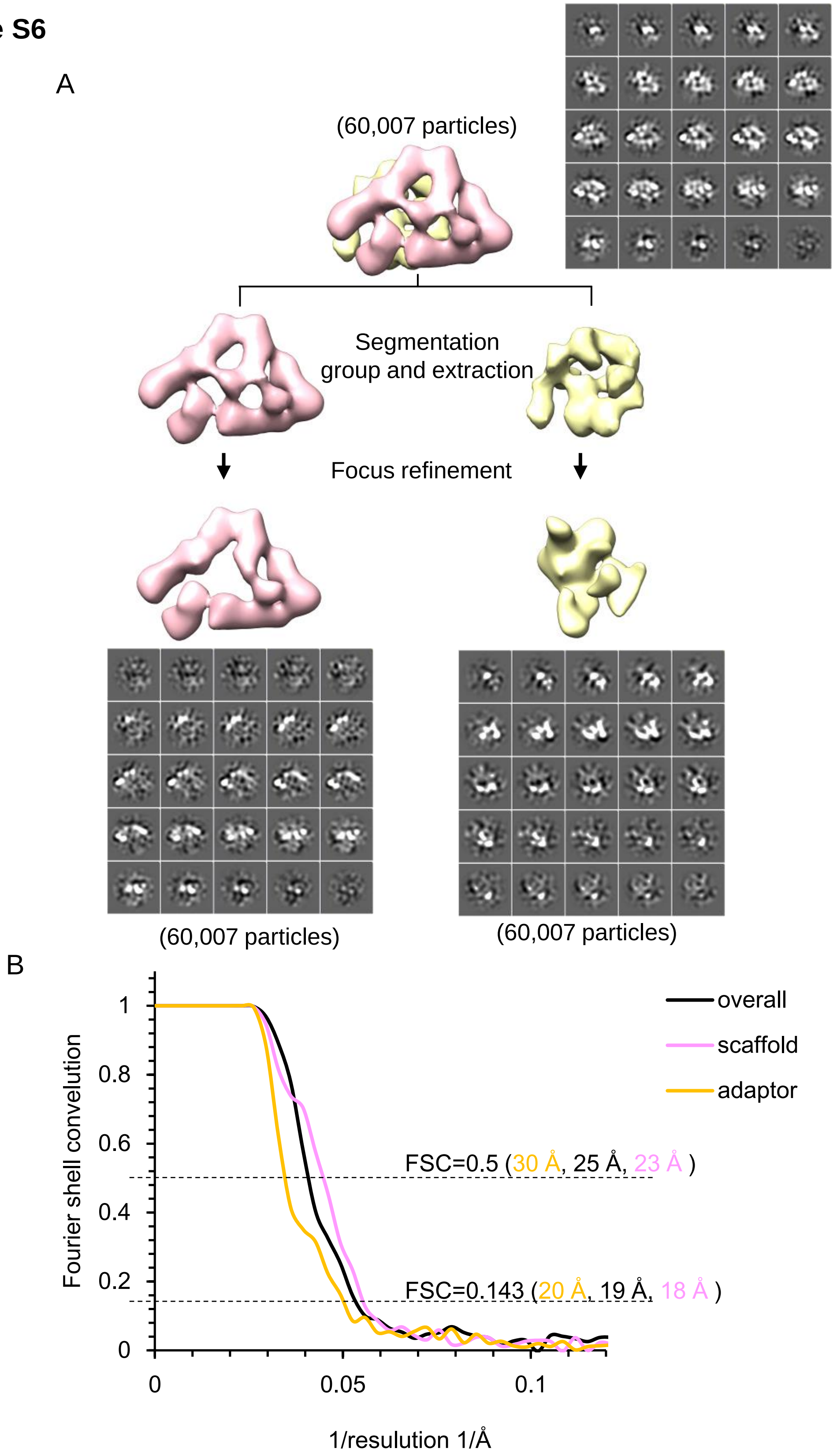

Figure S6. 3D reconstruction of the recombinant human coatomer. (**A**) "Focus" refinement process (see also **MATERIALS AND METHODS**). The iso-surface (displayed at levels corresponding to the volume of a single coatomer complex or as separated subcomplexes) and the ortho-slices are both displayed. (**B**) FSC curves of 3D reconstructions.

Figure S7

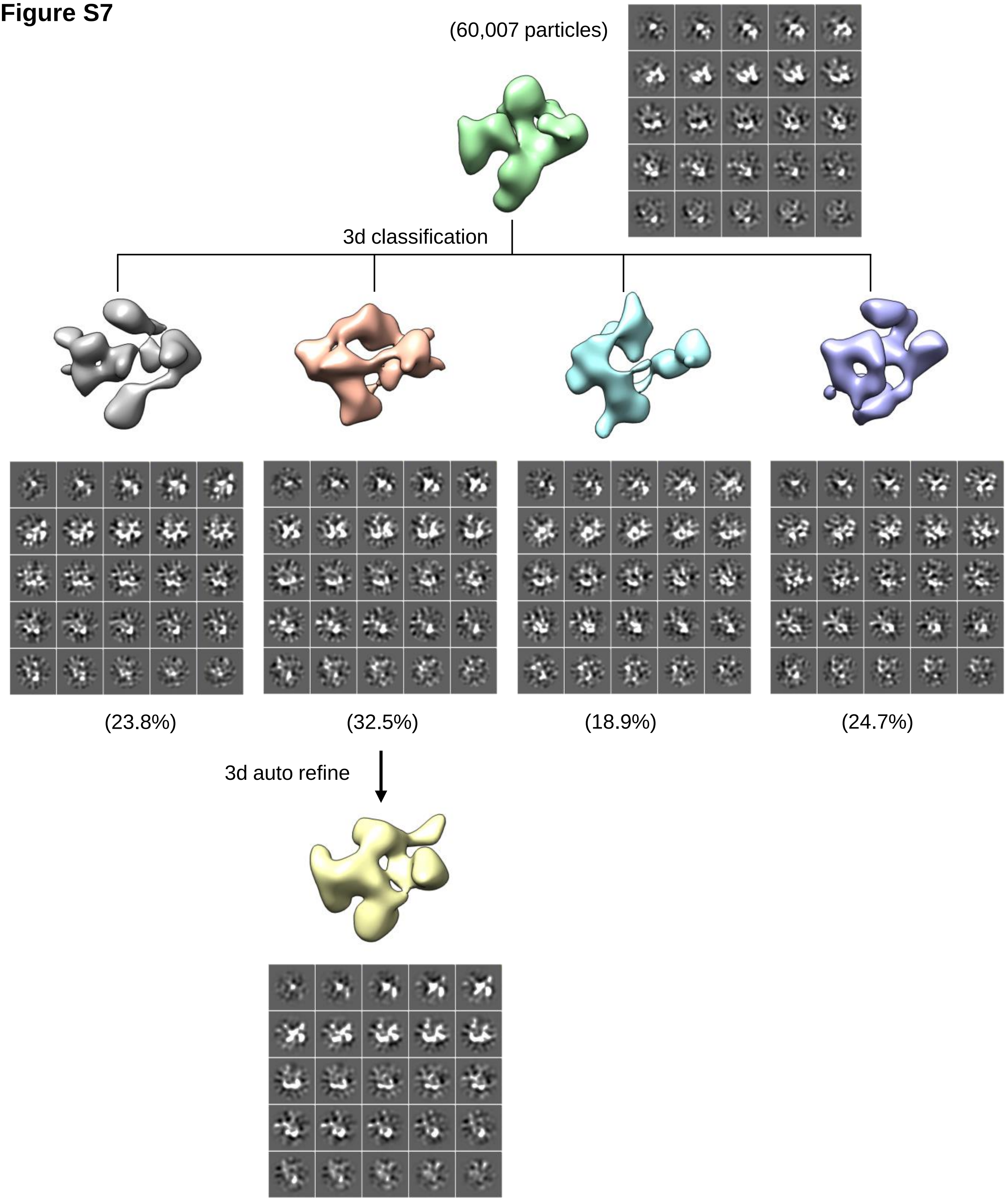

Figure S7. "Focus" classification of the adaptor F-subcomplex. The iso-surface (displayed at the same level corresponding to the volume of the F-subcomplex) and the ortho-slices are both displayed.
